# Predictive Factors for the First Recurrence of *Clostridioides difficile* Infection in the Elderly from Western Romania

**DOI:** 10.3390/medicina56090439

**Published:** 2020-08-29

**Authors:** Iosif Marincu, Felix Bratosin, Iulia Vidican, Bianca Cerbu, Mirela Turaiche, Livius Tirnea, Madalina Timircan

**Affiliations:** 1 Infectious Diseases, Victor Babes University of Medicine and Pharmacy, 300041 Timisoara, Romania; imarincu@umft.ro (I.M.); iulia.georgianabogdan@gmail.com (I.V.); ionitabiancaelena@yahoo.com (B.C.); mirela.turaiche@gmail.com (M.T.); liviustirnea@yahoo.com (L.T.); 2 Department of Gynecology, Victor Babes University of Medicine and Pharmacy, 300041 Timisoara, Romania; timircan.madalina@yahoo.com

**Keywords:** infection recurrence, geriatric population, infectious diseases, immunocompromised, *Clostridioides difficile*

## Abstract

*Background and objectives:* At present, Romania and parts of the European Union are facing an increasingly challenging public health problem consisting of nosocomial *Clostridioides difficile* infection (CDI), mostly in the elderly. Relapse cases have become more frequent, which present higher morbidity and mortality rates than the initial CDI infection. The aim of this study is to determine the predictive factors for recurrence, with the purpose of reducing the exposure of patients diagnosed with CDI, as well as aiming to initiate early treatment. *Materials and Methods:* In this retrospective descriptive study, we analyze a database from the First Department of Infectious Diseases at the Dr. Victor Babes Clinical Hospital for Infectious Diseases and Pulmonology in Timisoara, looking for patient history of CDI recurrences. We analyzed CDI recurrence in patients aged ≥65 years from 1 January 2016 to 31 December 2019, identifying 77 cases of CDI recurrence. The determination of predictive factors for recurrence involved the formation of a randomized control group, consisting of 74 patients aged ≥65 years who were diagnosed with *C. difficile* enterocolitis, but did not suffer a recurrence and survived ≥2 weeks after symptom onset. *Results:* Immunocompromised status, pre-existing gastrointestinal disease, and fever on initial hospitalization for CDI were all found to be significant independent positive predictive factors for the condition recurring in elderly Romanian patients. *Conclusions:* As the geriatric population in Romania grows, the national health system becomes increasingly overburdened, both from a financial standpoint and a human resources perspective. The analysis of factors predictive for CDI recurrence is, thus, of the utmost importance, particularly for the early identification of patients most at risk of CDI recurrence. Our findings could help physicians to identify recurrence early, consequently benefitting patients by a rapid intervention with a potential decrease in the associated complications and mortality.

## 1. Introduction

The incidence of *Clostridioides difficile* infection (CDI) has been increasing across Europe [1], greatly affecting healthcare facilities. The recurrence of CDI significantly increases morbidity, making effective treatment and disease management challenging.

Over the last decade, CDI has become an increasingly challenging condition for specialists to manage, due to its increasing incidence and severe complications. Since 2011, the annual incidence of CDI in both Romania and other European Union states has significantly increased [2], with ribotype 027 accounting for about 70% of total cases. Unfortunately, all Romanian hospitals are now affected, resulting in significant social and biomedical burden and decreased quality of life [3].

Symptomatic recurrence of CDI causes significant morbidity, and effectively treating this condition remains challenging. In addition, the transmission risk of *C. difficile* increases with disease recurrence. Recurrence rates have been reported to range from 5% to 50%, typically averaging around 20% [4]. 

The aim of this study was to determine predictive factors for the first recurrence of CDI in elderly patients (defined as individuals aged ≥65 years), with the underlying goal of improving the management of such cases. The early identification of high-risk patients could prevent recurrence altogether, thereby improving public health and minimizing the resultant economic consequences.

## 2. Materials and Methods

This paper details our retrospective, descriptive study, which evaluated data compiled by the First Department of Infectious Diseases at the Dr. Victor Babes Clinical Hospital for Infectious Diseases and Pulmonology in Timisoara, Romania. Cases of CDI recurrence involving elderly patients (defined as individuals aged ≥65 years) at this facility from 1 January 2016 to 31 December 2019 were analyzed. CDI recurrence was defined as cases in which symptoms reappeared 2–8 weeks after initially manifesting. The diagnosis of *C. difficile* infection was established by a history of diarrhea and *C. difficile* Toxin A/B stool assay results [5].

Data collected included demographic information (age, sex, country of origin), history of prior hospitalization, and medical and surgical history, as well as other diseases of the pulmonary, gastrointestinal, renal, and neurological systems, in order to assess the immune status of the patient. In this study, pre-existing gastrointestinal disease was considered in patients suffering of both acute and chronic gastrointestinal diseases, including inflammatory bowel disease, colon cancer, intestinal cancer, gastritis, necrotizing enterocolitis, mesenteric ischemia, and gastroenteritis of another cause than CDI. The immunocompromised group of patients in our sample consisted of HIV-infected patients, as well as patients undergoing immunosuppressant therapy after transplant surgery or autoimmune diseases. In addition, patients were monitored for sepsis, fever (≥38 °C), abdominal pain upon admission, and unfavorable disease evolution (i.e., death within 30 days of initial symptom onset).

From a total of 733 cases of elderly patients suffering CDI who had been hospitalized at our facility throughout the study period, we identified 77 cases (10.5%) with recurrence. Two recurrent cases (1.3%) hospitalized at the end of December 2015 and discharged in early 2016, were included in our analysis. In 2016, 25 patients (16.6%) were admitted; in 2017, 43 patients (28.5%); in 2018, 59 patients (39.1%); and, in 2019, 22 patients (14.6%).

Patients were also split by place of origin, that is, from rural or urban areas. This choice was made based on the social discrepancies between people inhabiting villages or cities in Romania, where access to safe and drinkable water, as well as the existence of bathrooms and sanitary equipment, is lacking significantly in rural areas.

Our tested variables included the treatment schemes followed in our clinic, ranging from oral metronidazole, vancomycin, mixed therapy (vancomycin and metronidazole), and other uncategorized drug schemes used only in particular cases.

Data were analyzed using the IBM SPSS version 20.0.0.0 software for Windows. A normality test was performed prior to applying the Student’s t-test, in order to compare means for continuous variable analysis, while the χ^2^ test was applied to compare proportions between recurrence and no-recurrence groups. Variables determined to be significant by univariate analysis were then included in a multivariate analysis. Correlations between the variables collected in our study were tested, in order to observe any associations that must be taken into consideration when making conclusions, and they were analyzed using Pearson’s correlation coefficient for continuous variables and Spearman’s correlation coefficient for ranked variables.

The final model, with independent predictors, was constructed by applying logistic regression with forwarding selection. Continuous data are presented numerically with percentages, while categorical data are presented using interquartile ranges (IQR). All data were tested maintaining a significance threshold of 0.05.

## 3. Results

**Figure 1:**
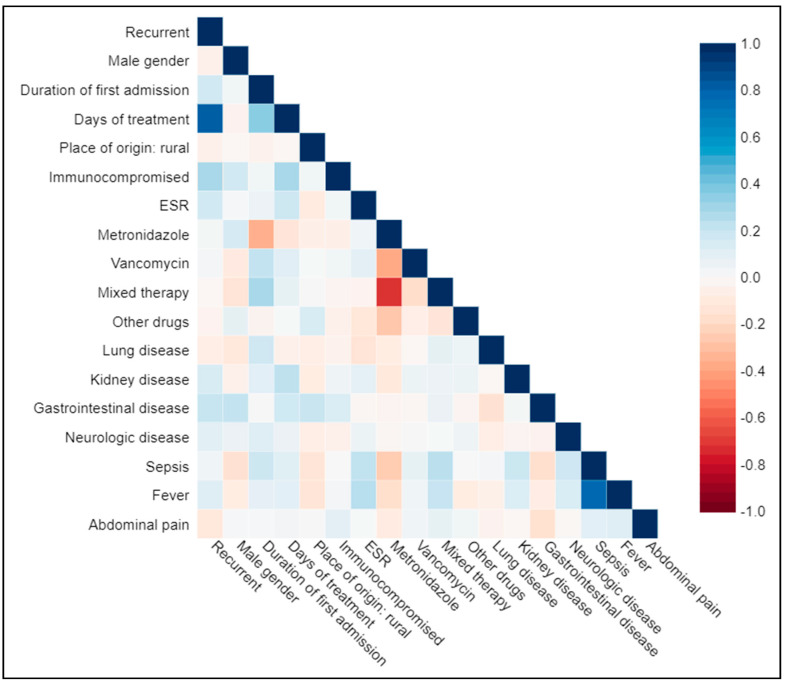
Correlation matrix.

The median length of hospitalization was 11 days for patients who did not suffer CDI recurrence, and 23 days for patients who did suffer a recurrence; this finding was significant (*p* = 0.04). No other statistically significant differences between control patients and recurrent cases were noted. The present study included a total of 151 patients, 77 of whom suffered CDI recurrence and 74 of whom did not. Gender was homogenously distributed among both groups (37 men and 40 women in the recurrence group; 32 men and 42 women in the control group). The median age of patients who suffered CDI recurrence was 76 years old, and that of patients who did not suffer recurrence was 75 years old. The correlation matrix for all variables tested is shown in Figure 1.

The total number of days of treatment was considered as the duration of hospitalization for CDI, as no patient was discharged before the infection had been successfully treated. The total length of treatment showed a strong and positive correlation with CDI recurrence (r > 0.8); however, the findings were not statistically significant. Immunocompromised status was positively correlated with CDI recurrence (r > 0.4). As expected, sepsis and fever had a strong positive correlation (r > 0.8). Oral metronidazole treatment had a strong negative correlation to the duration of first admission (r < −0.5), as well as negative correlations with vancomycin (r < −0.4), mixed therapy (r < 0.8), other drugs (r < −0.4), sepsis (r < −0.4), and fever (r < −0.2). From the correlation analysis, we observed that gastrointestinal disease was positively correlated to recurrent CDI (r > 0.3), male gender (r > 0.4), days of treatment (r > 0.3), and rural place of origin (r > 0.4).

We introduced the six statistically significant variables from our univariate analysis into our multivariate analysis, namely fever, immunocompromised status, associated digestive comorbidities, CRP level, potassium level, and serum sodium level. Sodium, potassium, and C-reactive protein levels significantly differed among the two patient groups (*p* = 0.029; *p* = 0.023; *p* = 0.048, respectively); however, the results were not found to be significant when evaluated by multivariate analysis. Death occurred in 20 patients (13.2%), 16 of which suffered a recurrence.

As found in Table 1, pre-existing gastrointestinal disease was discovered in 34 patients (44.2%) who suffered CDI recurrence, as compared with 18 patients (24.3%) of the control group; risk of recurrence for those with a pre-existing gastrointestinal condition was found to be three times greater (OR 3.307, CI: 1.506–7.261, *p* = 0.003). Immunocompromised patients were found to be associated with the highest risk of CDI recurrence (OR: 7.279, CI: 5.128–9.044 *p* = 0.007). The presence of fever at disease onset was found to be a statistically significant factor in the regression analysis; patients presenting fever on initial hospitalization for CDI were at double the risk of recurrence, as compared to patients without fever (OR 2.098, CI: 1.013–4.347, *p* = 0.046).

**Figure 2:**
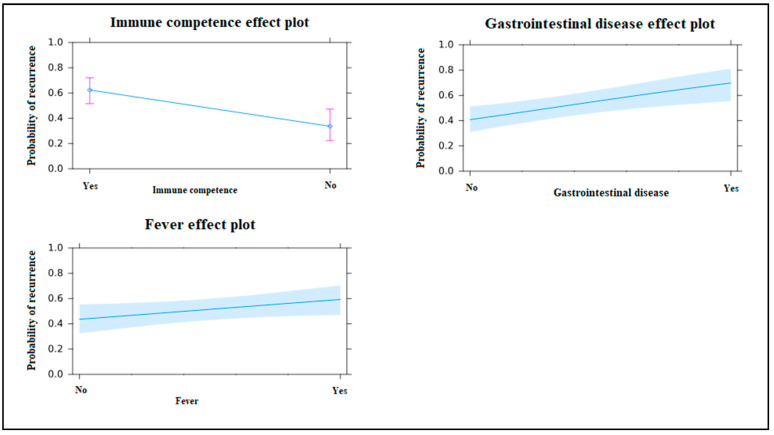
Independent predictive factors for first *C. difficile* enterocolitis recurrence.

Immunocompromised status, pre-existing gastrointestinal disease, and fever on initial hospitalization for CDI were all found to be independent positive predictive factors for recurrence of the condition (Table 2; Figure 2).

## 4. Discussion

### 4.1. Review of Literature

Interestingly, some risk factors previously identified as independent predictors of CDI recurrence among the general population were not identified as such in our study; for example, although the duration of hospitalization was associated with an increased risk of CDI recurrence in the study of Chopra [5], we did not find this variable to influence the disease course. The presence of fever on initial hospitalization was identified to also be an independent predictive factor for CDI recurrence. Fever was also identified to be a predictive factor in Chopra’s study, although not as an independent factor, but in bivariate analysis.

In a study by Khanafer, male gender and high C-reactive protein levels were reported to be associated with both CDI severity and a higher risk of recurrence; however, these findings were not confirmed by our present study [6].

Eyre et al. [7] evaluated a sample of 1678 patients and found that prior hospital admission, as well as hospitalization in gastroenterology wards, was associated with increased rates of recurrence. Similarly, we found that pre-existing gastrointestinal conditions were a predictive factor for CDI recurrence.

One particular aspect that might influence our findings, based on the data collected from the Dr. Victor Babes Clinical Hospital for Infectious Diseases and Pulmonology in Timisoara, is the endemic character of CDI in the hospital at Timisoara, as our department is the only one responsible for treating these cases [8]. However, another study [9] indicated that the risk of CDI recurrence was inconsistently correlated with the place of infection acquisition.

Several prior studies [9,10] have reported predictive factors for CDI recurrence. In the general population, increased risk of recurrence has been associated with increased age, leukocytosis (≥15,000 cells/µL), treatment with proton pump inhibitors after CDI onset or concomitant antibiotic treatment in addition to CDI therapy, severe/fulminant *C. difficile* enterocolitis, and antitoxin A-positivity with serum IgG < 1.29 units. Here, we aimed to determine independent factors for CDI recurrence in the elderly. In the same studies [10,11], immunocompromised status was observed to be a positive predictor for CDI recurrence, in agreement with our findings.

### 4.2. Limitations

The microbiology lab in our institution does not use a two-step method for CDI diagnosis (glutamate dehydrogenase followed by toxin assay), nor the highly accurate PCR test. Our method of diagnosis was the toxin test, which has a sensitivity of 88%, meaning that 12% of the tested patients may have been excluded due to false-negative results. Infection with the 027 virulent strain was not assessed as a risk factor for recurrence, as our institution does not use PCR testing to identify these particular cases.

Due to the retrospective design of our study, clinical data were manually collected from written patient histories. Missing information or inaccuracies were encountered, as data were obtained from several unstandardized sources. The fact that this study was conducted in a single center and that it evaluated only a limited sample size must be taken into account, concerning data heterogeneity.

Another limitation of the current research was the small sample size, which decreased the power of the study.

### 4.3. Future Perspectives

In this paper, we identified predictive factors for the first recurrence of CDI in elderly Romanian patients. Our findings provide a foundation for the further study of predictive factors for mortality in patients who suffer from CDI recurrence, especially considering that this patient population is characterized by an increased mortality rate. In addition, a more rigorous analysis of hospital data may allow for the development of a scoring system, which could help to identify the CDI cases at highest risk for recurrence.

## 5. Conclusions

The present study identified three independent predictive factors for the first recurrence of *C. difficile* infection, namely, the immunocompromised status, the presence of gastrointestinal disease, and fever on initial hospitalization.

*C. difficile* enterocolitis comprises a major public health issue worldwide. As the geriatric population in Romania grows, the national health system becomes increasingly overburdened, both from a financial standpoint and a human resources perspective. The analysis of factors predictive for CDI recurrence is, thus, of utmost importance, particularly for the early identification of patients most at risk for CDI recurrence. The importance of the results of the current study is that they contribute towards providing a means for identifying patients at risk for recurrence, in order to reduce their exposure to such risk factors.

## Figures and Tables

**Table 1:** Study sample demographic characteristics and comorbidities.

	Subtypes	Number (%) or Median (IQR)		Univariate Analysis (*p*-Value)	Multivariate Analysis (*p*-Value)
**Factor**		**No Recurrence**	**Recurrence**		
CDI		74 (49)	77 (51)		
Demographic data					
Gender	Male	32 (43.2)	37 (48.1)	0.252	
	Female	42 (56.7)	40 (51.9)		
Age		75 (9)	76 (11)	0.471	
Place of origin	Rural	41 (55.4)	39 (50.6)	0.293	
	Urban	33 (44.5)	38 (49.4)		
Past hospitalization or institutionalization	Yes	45 (60.8)	51 (66.2)	0.382	
	No	29 (39.1)	26 (33.8)		
Duration of CDI hospitalization		11 (7)	23 (10)	0.254	
**Comorbidities**					
Immunocompromised	Yes	37 (50)	55 (71.4)	0.01	0.007
	No	37 (50)	22 (28.5)		
Lung disease		27 (36.4)	23 (29.9)	0.592	
Kidney disease		28 (37.8)	40 (51.9)	0.437	
Gastrointestinal disease		18 (24.3)	34 (44.2)	0.001	0.01
Neurologic disease		31 (41.8)	40 (51.9)	0.796	
Sepsis		30 (40.5)	34 (44.2)	0.102	
Other disease(s)		74 (100)	77 (100)		
**Treatment**					
Metronidazole		45 (60.8)	48 (62.3)		
Vancomycin		4 (5.4)	7 (9.1)		
Metronidazole+Vancomycin		21 (28.3)	19 (24.7)		
Other(s)		4 (5.4)	3 (3.9)		
**Lab values**					
Leukocyte count (/µL)		9620 (4838)	9340 (9980)	0.693	
ESR (mg/L)		30 (45)	35 (38)	0.762	
CRP (mg/L)		42 (85)	81 (121)	0.048	
Sodium (mmol/L)		135 (7)	138 (7)	0.029	
Potassium (mmol/L)		3.5 (1)	3.7 (1)	0.023	
**Symptoms**					
Abdominal pain		74 (100)	75 (97.4)	0.637	
Fever (≥38 °C)		74 (100)	41 (53.2)	0.001	<0.0001
Death within 30 days		4 (5.4)	16 (20.8)		

IQR = interquartile ranges; CDI = *Clostridioides difficile* infection; ESR = Erythrocyte Sedimentation Rate; CRP = C-reactive Protein.

**Table 2:** Independent predictive factors for the first recurrence of *C. difficile* infection.

	OR	95% CI	*p*-Value
Immunocompromised	7.279	5.128–9.044	0.007
Presence of gastrointestinal disease	3.307	1.506–7.261	0.003
Fever	2.098	1.013–4.347	0.046
